# A Case of Reactivated Disseminated Coccidioidomycosis in an Elderly Patient

**DOI:** 10.7759/cureus.94735

**Published:** 2025-10-16

**Authors:** Gabriela Rodriguez, Roya Mojarrad, Diana Sarkisyan

**Affiliations:** 1 Department of Medicine, University of California Los Angeles Health, Simi Valley, USA

**Keywords:** coccidioidomycosis, disseminated disease, elderly people, fungal infection, reactivated disease, san joaquin valley fever, soft-tissue infection, soft tissue mass

## Abstract

Reactivation of extrapulmonary disseminated coccidioidomycosis infection is rare. When it does happen, it often happens soon after the primary infection. Reactivation of latent disease is usually seen in patients who later become immunocompromised. Here we discuss a case of an 88-year-old male patient with a distant history of extrapulmonary coccidioidomycosis infection 50 years prior, who developed reactivated coccidioidomycosis presenting as a soft tissue mass in his arm and new polyarthritis. Despite rapid initiation of fluconazole treatment, the patient struggled with his recovery, which was complicated by deep vein thrombosis (DVT) and deconditioning at his age. We believe our patient's age, gender, and comorbidities were contributing factors for his reactivation and disseminated disease. This case illustrates the importance of having a high index of suspicion for reactivation of disease despite a long latent period.

## Introduction

Coccidioidomycosis is a fungal infection, caused by Coccidioides species, that is endemic to the Southwest United States and Northern Mexico [1,2]. It is acquired through the inhalation of spores present in the soil of endemic areas. Coccidioidomycosis has a wide range of clinical manifestations, with pulmonary infection being the most common [2]. Primary pulmonary disease can range from mild respiratory illness to systemic disease that can resolve spontaneously over a period of weeks to months [3]. A minority of cases are complicated by persistent pulmonary disease or by disseminated extrapulmonary disease [3]. Disseminated extrapulmonary disease is rare, affecting 1% to 3% of all cases [1,2]. Dissemination can affect almost any organ in the body, but most commonly involves the meninges/central nervous system (CNS), skin, bone, and joints [1,2]. Disseminated infections rarely resolve without treatment and can be fatal in the absence of systemic antifungal therapy [3]. Diagnosis is made serologically, by histopathologic examination, or from tissue culture [1]. The mainstay of treatment for most patients is azoles [1,2,4]. Patients who are of certain ethnic groups, are immunocompromised, or are pregnant are at a higher risk for disseminated disease and reactivation of the latent infection [1,4]. It is important to recognize that while infection gives the patient lifelong immunity, it can remain dormant for years and later reactivate if the patient becomes immunocompromised [5,6]. This case examines the reactivation of Coccidioides 50 years after the initial infection and looks into the risk factors for disease reactivation in a patient who appeared to be immunocompetent.

## Case presentation

An 88-year-old Hispanic male patient from Southern California presented with 10 days of pain, redness, and swelling of his left wrist and right knee. About two months prior, he had noticed an enlarging soft tissue mass on the back of his right upper arm. The mass had been present for about a year but had recently begun growing and becoming uncomfortable. He reported a distant history of coccidioidomycosis infection of his right elbow, which was treated 50 years ago with antifungals and surgical debridement. He denied any pulmonary involvement during his primary infection.

On review of systems, he reported mild fever and denied any chills, night sweats, cough, shortness of breath, or trauma. His past medical history included hypertension, type 2 diabetes mellitus, ischemic cardiomyopathy, aortic stenosis, and atrial fibrillation. His surgical history included a coronary artery bypass graft and transcatheter aortic valve replacement.

On examination, the patient’s temperature was 37.9˚C, pulse was 71 beats per minute, respiratory rate was 16, and blood pressure was 114/71 mmHg. His left wrist and right knee were visibly swollen, erythematous, warm to the touch, and mildly tender to palpation. He had notable edema of his entire right lower leg, extending from his knee down to his foot. He had a large fluctuant mass on the posterior aspect of his right upper arm, which was non-tender and non-erythematous. He was referred to the emergency room for urgent evaluation for disseminated coccidioidomycosis, given his history and polyarticular presentation. His labs showed an elevated serum Coccidioides antibody-complement fixation titer of 1:32 (reference range <1:2).

**Figure 1:**
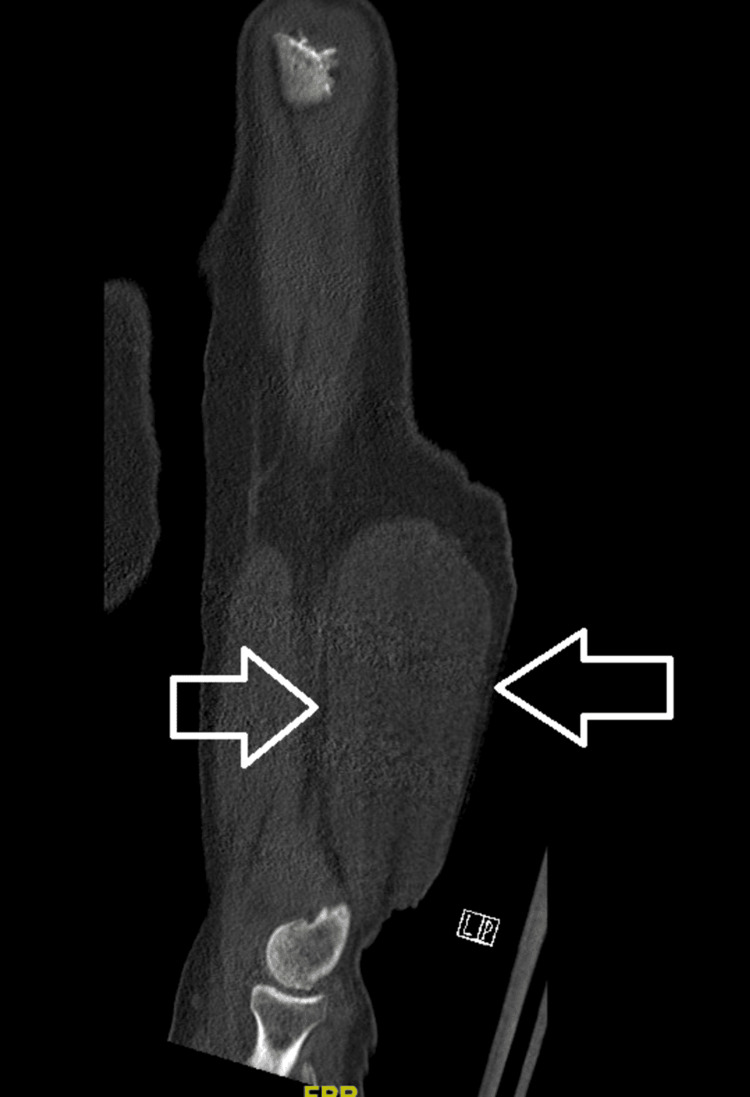
CT scan of the right human humerus The scan shows a 12 x 6.6 x 3.3 cm ovoid fluid-attenuating mass/collection in the posterolateral mid to distal arm located deep to the fascia with mass effect on the triceps. There was no periosteal reaction.

A computerized tomography (CT) scan of his right humerus showed an ovoid fluid-attenuating mass/collection in the distal portion of the arm located deep to the fascia with mass effect on the triceps, as seen in Figure 1.

There was no involvement of the surrounding bone noted in the CT scan. He underwent aspiration of the right arm mass, which showed a brown, cloudy fluid. The aspirate was sent for analysis, and showed an elevated white blood count consisting of mostly segmented neutrophils as seen in Table 1.

**Table 1:** Analysis of the right arm mass aspirate

Parameter	Result
Color	Brown
Clarity	Cloudy
White blood cell count	13,710 cells/mm^3^
Red blood cell count	720 cells/mm^3^
Segmented neutrophils, %	91%
Lymphocytes, %	2%
Monocytes, %	2%
Eosinophils, %	5%
Basophils, %	0%

Cytology from the aspirate showed 'rare fungal elements consistent with coccidioidomycosis.' Fluid cultures eventually grew Coccidioides. He had a duplex ultrasound of his right leg which showed a non-occlusive deep vein thrombosis (DVT) of his femoral vein.

The patient was diagnosed with disseminated coccidioidomycosis and discharged on fluconazole 600 mg daily. Magnetic resonance imaging (MRI) of his right humerus was ordered to evaluate the joint space for any effusion, destruction to bony structures, assess for abscess, and assess the surrounding tissue for surgical planning. The patient declined an MRI due to an implantable cardioverter-defibrillator and pacemaker incompatibility, so further aspiration of the right arm mass and debridement were deferred to the outpatient setting. The patient suffered the complication of right lower extremity DVT while on low-dose apixaban (Eliquis). His Eliquis dose was increased at discharge.

## Discussion

Coccidioides infections manifest broadly, with 60% of the cases being asymptomatic and 40% having a mild respiratory infection [1,2,4]. Approximately 1% of total infections cause disseminated disease [2]. Infection involving any other site that is not pleural/pulmonary is considered disseminated [2]. Disseminated disease is spread hematogenously and most commonly involves the skin, bones, joints, and central nervous system/meninges [2,5]. The initial sign of reactivated disease in our patient was a soft tissue mass that slowly grew near the site of primary infection (right elbow). It did not seem to be causing any involvement of the right elbow at the time of reactivation. The infection eventually disseminated further, causing synovitis of his right knee and left wrist. Often, soft tissue infections and synovitis occur in association with osteomyelitis, but this was not seen in our case.

All cases of extrapulmonary coccidioidomycosis should be treated as they seldom resolve on their own; this is in contrast to pneumonic disease, where mild disease may not require treatment [4]. A majority of disseminated infections with musculoskeletal involvement require aspiration or surgical intervention along with systemic antifungal therapies [1,2,4]. The duration of treatment for disseminated disease varies with each case but is often recommended for three years [5]. Patients with multiple recurrences, immunosuppression, or other comorbidities may require longer treatment [2]. Our patient’s treatment was limited due to his increasing frailty and physical deconditioning, and an inability to undergo further imaging for surgical planning. His disease remained controlled with fluconazole for two years until he ultimately succumbed to his heart failure.

After an initial coccidioidal infection, there appears to be lifelong immunity, and reinfection does not occur [6]. Most cases of reactivated disseminated disease were seen within two months of the primary infection [3,4]. It is important to remember that Coccidioides can remain dormant for years and reactivate later, causing disseminated disease in those who subsequently become immunocompromised [5,6]. Those with the highest risk for severe or disseminated disease include those of Filipino and African-American descent, later stages of pregnancy, and persons with diseases or on therapies that cause cellular immunodeficiencies [1,3,5]. These immunosuppressed patients range from those infected with human immunodeficiency virus (HIV), those with genetic mutations to those receiving antirejection medications, high-dose steroids, chemotherapy, or other immunomodulating therapies used in the treatment of many rheumatologic or inflammatory bowel conditions [4]. Diabetes has been known to portend more severe primary pulmonary disease and is also seen as a risk factor for disseminated disease [4,5]. For patients with coccidioidal pneumonia and concurrent diabetes or who are frail from age or comorbidities, prompt treatment is recommended, compared to observational treatment for immunocompetent patients with mild or resolving pneumonia [4]. In this case, we saw the reactivation of dormant Coccidioides 50 years after primary infection, despite our patient not being on any immunosuppressive medications or having any apparent immunosuppressing conditions.

Although coccidioidomycosis can affect all age groups, it is most commonly seen in adults, and it is also more common among men than women [7]. Advanced age was always thought to be a common risk factor for coccidioidomycosis infection in part due to age-related immune dysfunction [7,8]. This is mainly characterized by a decrease in cell-mediated immunity. A robust cell-mediated immunity is the primary defense against coccidioidomycosis [5,8]. Several studies have investigated advanced age (above 60 years) as a risk factor for infection, but many have been inconclusive in showing it as an independent factor. One retrospective medical record review of coccidioidomycosis cases in Arizona found no significant difference in disease manifestations between patients ≥60 years of age and those <60 years old, when controlling for comorbid conditions. The study found immunosuppression to be the only factor associated with severe disease, dissemination, or death, regardless of age [7,8]. Another case-control study aimed to examine the risk factors associated with the increased incidence of coccidioidomycosis in the elderly, specifically in Arizona. They found that after controlling for the duration of residence, those of male sex, active smokers, corticosteroid use, or diagnosed with congestive heart failure or active cancer, were found to be independently associated with developing symptomatic coccidioidomycosis, when compared to geographically-matched groups [9]. In the review of this patient's echocardiograms before and after the reactivation of infection, we observed that his heart failure had worsened. His routine echocardiogram, done two months prior to his presentation, showed an ejection fraction of 46%. A repeat echocardiogram done three months after the reactivated infection showed an ejection fraction of 31%. We believe that our patient's advanced age and comorbidity of congestive heart failure led to an impaired immune response, which in turn led to the reactivation of Coccidioides. 

## Conclusions

This case demonstrates the reactivation of coccidioidomycosis infection in a patient without clear immunosuppression, over 50 years after the primary infection. While there is still no direct link between age and comorbid conditions as confirmed risk factors for reactivation of Coccidioides, we believe those factors, along with male sex, contributed to this patient's reactivation. More research is needed to help identify elderly patients who are at high risk for reactivation of coccidioidomycosis. This case illustrates the high index of suspicion one must have in an immunocompetent patient with a distant history of primary disseminated infection. Developing more preventive strategies and early recognition in the elderly population may help minimize disease reactivation.
